# Single-Cell RNA Sequencing Reveals Tissue Compartment-Specific Plasticity of Mycosis Fungoides Tumor Cells

**DOI:** 10.3389/fimmu.2021.666935

**Published:** 2021-04-21

**Authors:** Katharina Rindler, Wolfgang M. Bauer, Constanze Jonak, Matthias Wielscher, Lisa E. Shaw, Thomas B. Rojahn, Felix M. Thaler, Stefanie Porkert, Ingrid Simonitsch-Klupp, Wolfgang Weninger, Marius E. Mayerhoefer, Matthias Farlik, Patrick M. Brunner

**Affiliations:** ^1^ Department of Dermatology, Medical University of Vienna, Vienna, Austria; ^2^ Department of Pathology, Medical University of Vienna, Vienna, Austria; ^3^ Division of General and Pediatric Radiology, Department of Biomedical Imaging and Image-guided Therapy, Medical University of Vienna, Vienna, Austria; ^4^ Department of Radiology, Memorial Sloan Kettering Cancer Center, New York, NY, United States

**Keywords:** single-cell RNA sequencing, cutaneous T-cell lymphoma (CTCL), mycosis fungoides, tissue resident memory T cells, central memory T cells

## Abstract

Mycosis fungoides (MF) is the most common primary cutaneous T-cell lymphoma. While initially restricted to the skin, malignant cells can appear in blood, bone marrow and secondary lymphoid organs in later disease stages. However, only little is known about phenotypic and functional properties of malignant T cells in relationship to tissue environments over the course of disease progression. We thus profiled the tumor micromilieu in skin, blood and lymph node in a patient with advanced MF using single-cell RNA sequencing combined with V-D-J T-cell receptor sequencing. In skin, we identified clonally expanded T-cells with characteristic features of tissue-resident memory T-cells (T_RM_, *CD69^+^CD27^-^NR4A1^+^RGS1^+^AHR^+^*). In blood and lymph node, the malignant clones displayed a transcriptional program reminiscent of a more central memory-like phenotype (*KLF2^+^TCF7^+^S1PR1^+^SELL^+^CCR7^+^*), while retaining tissue-homing receptors (CLA*, CCR10*). The skin tumor microenvironment contained potentially tumor-permissive myeloid cells producing regulatory (*IDO1*) and Th2-associated mediators (*CCL13, CCL17, CCL22*). Given their expression of *PVR, TNFRSF14* and *CD80/CD86*, they might be under direct control by *TIGIT^+^CTLA4^+^CSF2^+^TNFSF14^+^* tumor cells. In sum, this study highlights the adaptive phenotypic and functional plasticity of MF tumor cell clones. Thus, the T_RM_-like phenotype enables long-term skin residence of MF cells. Their switch to a T_CM_-like phenotype with persistent skin homing molecule expression in the circulation might explain the multi-focal nature of MF.

## Introduction

Primary cutaneous T-cell lymphomas (CTCL) comprise a heterogeneous group of peripheral non-Hodgkin’s lymphomas (1, 2), with mycosis fungoides (MF) as their most frequent clinical entity. Usually, MF shows an indolent course with stable or only slowly progressing lesions confined to the skin, resulting in an overall 5-year survival rate of 70-80% (3). In line with this biological behavior, malignant T-cells show many features consistent with non-migratory, skin-resident memory cells (T_RM_) (4). Yet, in some patients, initial patches and plaques develop into tumors, eventually leading to systemic disease, with potential involvement of lymph nodes, blood, bone marrow and internal organs. Increasing expression of exhaustion markers by infiltrating CD4^+^ and CD8^+^ T-cells (5), and a shift from a Th1 towards a more Th2-like immune microenvironment are currently assumed to interfere with effective anti-tumor immune responses during progression of disease (6). However, exact mechanisms remain to be elucidated, and only little is known about the actual processes facilitating dissemination of tumor cells to extracutaneous sites. Using high-throughput TCR-β and TCR-γ sequencing, Kirsch et al. demonstrated seeding of a clonal population of malignant T_RM_ to distant skin sites and the peripheral blood, and found the malignant clone to descend from a mature T-cell according to the number of rearranged TCR-γ genes (7, 8). Recently, this hypothesis has been challenged by data from copy number aberration and TCR clonotype analyses using whole exome and whole transcriptome sequencing, that suggest the presence of oligoclonal malignant T-cells in MF lesions (9, 10). Single-cell RNA sequencing (scRNA-seq) combined with single-cell V-D-J sequencing now bears the potential to shed new light on these incongruities. By simultaneous determination of clonality through sequencing of the TCR-α and β chains and analysis of differentially expressed genes, malignant and tumor infiltrating cells can be easily discriminated, and their interaction can be readily analyzed (11). Here we profiled skin, lymph node and peripheral blood from a patient with stage IVB MF at single cell resolution. We were able to consistently detect the malignant clone and assess its properties and dynamic interplay with the microenvironment specific to each of these body compartments, demonstrating considerable tumor cell plasticity spanning from T_RM_ characteristics in skin to more T_CM_-like properties in blood and lymph node.

## Methods

### Patient Recruitment

The study was conducted under a protocol approved by the Ethics Committee of the Medical University of Vienna, Austria (EK 1360/2018). After written informed consent, a 65 years old Caucasian woman with stage IVB (T3 N3 M1 B1) Mycosis fungoides (MF) was included. At time of sampling, the patient did not receive disease-specific treatment. Routine laboratory investigations revealed an LDH of 322U/L (normal range <250U/L) and a CRP value of 11.47mg/dL (normal range <0.5mg/dL).

### Sample Acquisition and Cell Sorting

A 6mm skin punch biopsy from an MF lesion was obtained from the right flank. The biopsy was minced with scalpels and digested in collagenase IV (0,5U/ml, Worthington) for 30min at 37°C to obtain a single cell suspension. Lymph node cells were obtained from a small fraction of a diagnostic biopsy from a pathologically enlarged axillary lymph node. PBMC were prepared by Ficoll^®^ Paque density gradient centrifugation (GE Healthcare). All cell suspensions were frozen at -80°C until further processing. Cell suspensions were thawed and stained with CD7 FITC, CD4 PE, CD3 APC, CD45 ECD and 7-AAD. In the case of skin and lymph node, viable CD45^+^CD3^+^CD4^+^ T-helper cells, other CD45^+^ cells, and CD45-negative cells were sorted on a FACS Aria III (BD Biosciences) at a ratio of 1:2:1. In the case of PBMC, viable CD3^+^CD4^+^ helper T-cells and remaining CD45^+^ cells were sorted in a ratio of 1:2. Immediately after sorting, cells were subjected to scRNA-seq processing (10X Genomics, Pleasanton, CA) according to the manufacturer’s instructions.

### Droplet-Based, Single-Cell RNA Sequencing

T-cell receptor (TCR) sequencing and 5’ gene expression sequencing was performed using the Chromium Controller and Single Cell 5’ Library & Gel Bead Kit (10x Genomics) according to the manufacturer’s protocol. Sequencing was performed using the Illumina NovaSeq 4000 platform and the 150bp paired-end configuration.

### Data Analysis

RNA sequencing files were preprocessed with the Cell Ranger software from 10x Genomics (version 3.0.2, RRID: SCR_017344). After demultiplexing with the Cell Ranger command ‘mkfastq’, reads from each transcriptome library were aligned to the human reference genome assembly ‘refdata-cellranger-GRCh38-3.0.0’ using the ‘cellranger count’ pipeline. The pipeline generated both a raw UMI count matrix, including counts for all the droplets, and a filtered UMI count matrix, including only those droplets which are likely to contain at least one cell.

Reads from TCR receptor libraries were assembled using the ‘cellranger vdj’ pipeline in the reference-assisted mode using the vdj_GRCh38_alts_ensembl-3.1.0 reference version. The pipeline also annotated the assembled contigs according to the reference and grouped cells into clonotypes.

Filtered gene expression matrix, TCR amino acid sequences and clonotypes were then used for secondary analysis with R version 3.6.3 (2020–02–29).

### Secondary Analysis

Seurat package (version 3.1.4, RRID: SCR_016341) was applied to perform quality control and integrate all samples. The filtering criteria applied to each cell were the number of genes expressed (between 200 and 4000) and mitochondrial gene percentage (less than 12%) in order to discriminate for multiplets and dead cells leaking mRNA but include cell types with naturally higher mitochondrial content (such as macrophages). All cells not satisfying these criteria were discarded. Following QC, data from TCR receptor sequencing and transcriptome sequencing were merged by adding clonotype frequency and CDR3 amino acid sequences to the metadata column of the Seurat objects. All samples were aligned with the standard integration pipeline, as recommended by the Seurat developers (12, 13). Briefly, gene expression counts were log-normalized and 2,000 variable features were selected individually for each sample, and used to find integration anchors, and for principal component analysis. Based on explained variance by each principal component (elbow plot), we selected the first 22 principal components as input for dimension reduction and clustering using the Louvain algorithm at a resolution of 0.6. Clusters were visualized in two-dimensional space by Uniform Manifold Approximation and Projection (UMAP). The corresponding cell types of clusters were annotated by finding cluster markers with the “FindAllMarkers” command and running the SingleR package (1.0.5) (14). Differential gene expression (logFC>|0.25|, adjusted p-value<0.05) was calculated using the FindMarkers command and the Wilcoxon Rank Sum Test. P-values were adjusted for multiple comparisons with Bonferroni correction. We used scran package to find droplets containing more than one cell (15). The applied approach simulates thousands of doublets by adding together two randomly chosen single cell profiles. For the doublet score calculation cell clustering including the set randomly generated doublets was performed. Then for each cell of the original dataset the number of simulated doublets in their neighborhood was recoded and used as input for score calculation. We used 200 nearest neighbors for each cell. Doublet score was log10 of the ratio between simulated doublet cells and total number of neighbors taken into consideration for each cell.

Calculation of cell cycle scores was performed as implemented in the Seurat package, where gene expression of cell cycle marker genes was combined to a score. The score consisted of 43 genes primarily expressed in G1/S and 55 primarily expressed in G2/M, described in more detail by Tirosh I et al. (16).

### Monocle Analysis

Trajectories and Pseudotime were calculated using Monocle 2 version 2.13 (RRID: SCR_016339) (17, 18). Briefly, malignant cells (i.e. TRB1+ or TRB2+ cells) of clusters TC-1 and TC-3 of the filtered ‘Seurat’ object were converted to a CellDataSet and size factors and dispersions were estimated. Low quality cells and genes expressed in fewer than 10 cells were removed and unsupervised clustering was performed after “tSNE” dimension reduction (to 15 dimensions) and by specifying “tissue” as model formula for batch effect removal.

As model formula input for the first step of trajectory construction, differentially expressed genes between the tissues skin, blood and lymph node were used. In the next step dimensions were reduced with DDRTree and cells were ordered along the constructed trajectory and colored according to pseudotime, tissue of origin or cell cycle phase. For each branching point of the trajectory, we did branched expression analysis modelling (BEAM) (19) and genes with *q*‐value <0.00001 were displayed in a heat map, after eliminating ribosomal and mitochondrial genes.

### Copy Number Variation Analysis

Copy number variation analysis was performed using inferCNV of the Trinity CTAT Project (https://github.com/broadinstitute/inferCNV). The software compares gene expression values across genomic positions between case and control cells to identify genomic regions with consistent higher or lower signal intensities. We used a window of 201 for moving average smoothing and minimum of 5 cells per gene to include the corresponding gene into the analysis. CNVs were predicted with a Hidden Markov Model. We used a 6-state CNV model, attempting to predict either complete loss, loss of one copy, no change, addition of one copy, addition of two copies or addition of more than two copies.

### Resource Availability - Data and Code Availability

The 10X Genomics datasets generated during this study are available *via* Gene Expression Omnibus GSE165623. The published article includes detailed descriptions on how publicly available coding pipelines were used during this study. The exact code is available from the corresponding author upon request. Further information and requests for resources and reagents should be directed to the Lead Contact, Patrick M. Brunner (patrick.brunner@meduniwien.ac.at).

## Results

### Patient Characteristics

**Figure 1:**
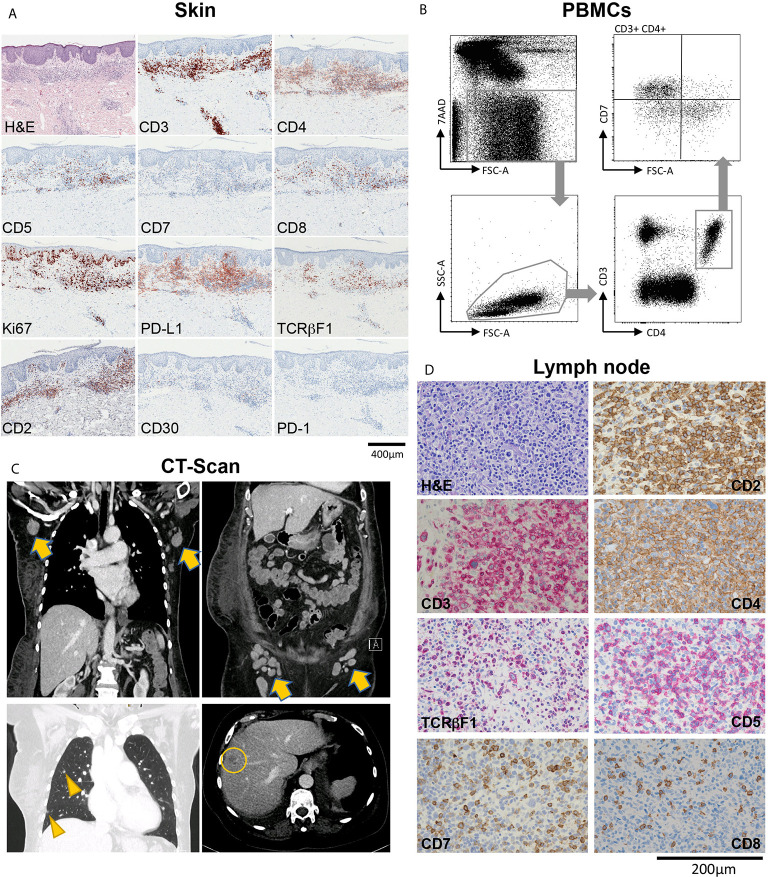
Overview of a patient with advanced-stage mycosis fungoides (MF) with skin, blood, lymph node and internal organ involvement. **(A)** Histopathological evaluation of a skin biopsy showing an inflammatory infiltrate highly suspicious of MF, with T-cells showing partial loss of CD5, and total loss of CD7. **(B)** Flow-cytometric evaluation of peripheral blood mononuclear cells (PBMCs) showing a large T-cell population with CD7 loss within the CD3+CD4+ helper cell compartment. **(C)** Contrast-enhanced CT chest, abdomen and pelvis showing pathologically enlarged axillary and inguinal lymph nodes (arrows), and lesions to the lung (arrow heads) and the liver (circle) suspicious of CTCL manifestations. **(D)** Histopathological characterization of a diagnostic biopsy of an axillary lymph node, confirming CTCL involvement.

A 65-year-old female patient suffering from MF had been in care at our department for a total of 14 years. She was originally referred in 2006 with stage IIB (T3 N0 M0 B0) disease with patches, plaques and tumors on her head and lower extremities. The Modified Severity Weighted Assessment Tool (mSWAT) showed a severity score of 8.2, indicating generally limited skin involvement. Until 2012, treatments consisted of topical corticosteroids, involved site radiation therapy (ISRT), systemic retinoid treatment (bexarotene), total skin electron beam (TSEB) radiation therapy, and gemcitabine chemotherapy, which led to temporary remission of disease. After seven years of loss to follow up, she returned to our clinic in 2019, presenting with generalized patches, plaques and tumors, and an mSWAT of 108. Skin histopathology revealed a dense subepidermal infiltrate of small to medium sized lymphocytes positive for CD2, CD3, CD4, PD-L1 and TCRβF1, partial positivity for CD5, and negative stainings for CD7, CD30 and PD-1. Ki67 staining showed a proliferation index of approximately 20% (**Figure 1A**). In peripheral blood, the absolute leukocyte count was 7400/µl (normal range 4000-10,000), with 885 T-cells (CD3) per microliter (data not shown). 13% of blood T-cells (ie 1.6% of all leukocytes) showed an aberrant phenotype of high forward scatter (FSC-A), elevated CD4 and decreased CD7 levels (**Figure 1B**), with negativity for CD5 and CD30 (data not shown). Contrast-enhanced computed tomography (CT) of the chest, abdomen and pelvis revealed pathologically enlarged axillary, para-aortic, iliac and inguinal lymph nodes, as well as hepatic and pulmonary lesions suspicious of CTCL manifestations (**Figure 1C**). Routine histologic examination of a right axillary lymph node showed paracortical infiltration of conspicuous atypical small-to-medium sized lymphocytes, positive for CD2, CD3, CD4 and TCRβF1, with partial loss of CD5 and CD7 (**Figure 1D**), consistent with lymph node involvement of MF (Dutch grade N3). These cells were negative for PD-1, with a proliferation index of up to 50% (data not shown). There was no histological evidence for large cell transformation (LCT) neither in the skin nor the lymph node. In sum, the patient was diagnosed with stage IVB (T3 N3 M1 B1) disease.

### Skin, Blood and Lymph Node Profiling Using Single-Cell RNA Sequencing

**Figure 2:**
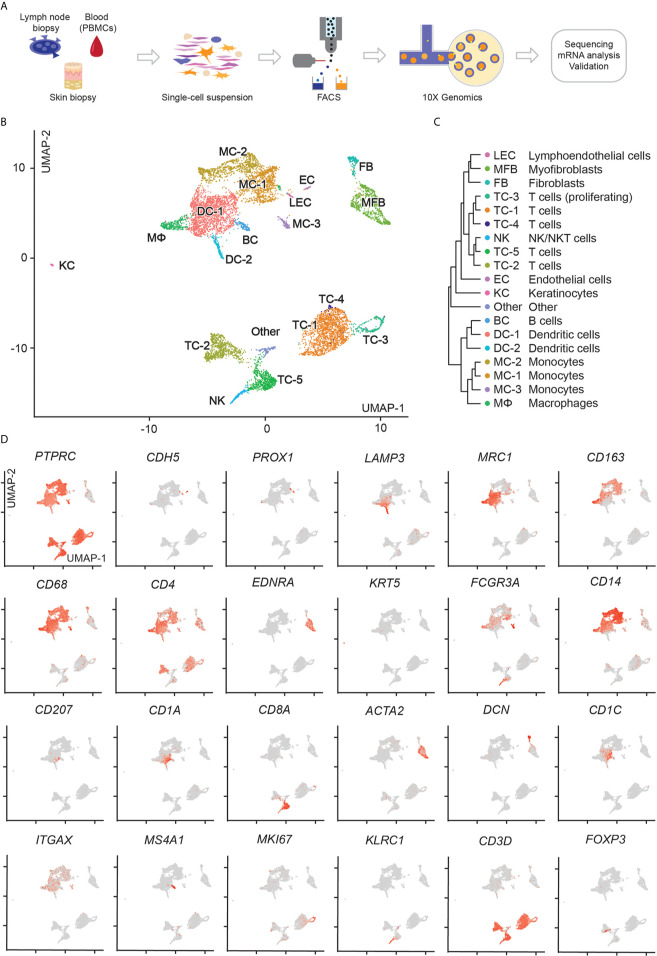
scRNA-seq workflow and map of isolated cells from lymph node, blood, and skin. **(A)** Outline of steps for single cell profiling of trancriptomes from different tissues. **(B)** UMAP of 9,028 cells integrated from lymph node (1,215), blood (3,301), and skin (4,512) samples according to similarity of their transcriptome, resulting in 19 different color-coded clusters. **(C)** Unsupervised hierarchical clustering showing relatedness of cell clusters (average gene signatures; correlation distance metric and average linkage). **(D)** Combined feature plots of all samples showing expression distribution for canonical markers. Intensity of normalized expression for each cell is color-coded (red) and overlaid onto UMAP plots.

To better understand tumor cell characteristics across tissues in this MF patient, we performed single-cell RNA sequencing (scRNA-seq) of cells sorted from involved skin, blood and lymph node, as outlined in **Figure 2A**. Sequencing data were filtered for low-quality cells and normalized, yielding 1,215 lymph node, 3,301 blood and 4,512 skin cells (**Table S1**). Clustering of these three compartments followed by visualization using uniform manifold approximation and projection for dimension reduction (UMAP) (20) revealed 19 distinct cell clusters (**Figures 2B, C**). We assigned cell identities on the basis of canonical markers (**Figure 2D**) and identified the top 10 upregulated genes (according to average log fold change and smallest adjusted p-value) for each cluster in comparison to all other clusters (**Figure S1** and **Table S2**). We found *PTPRC*/CD45-negative cells consistent with endothelial cells (EC: *CDH5*/VE-cadherin), lymphatic endothelial cells (LEC: *PROX1*), fibroblasts (FB: *DCN*,), keratinocytes (KC: *KRT5*) and myofibroblasts (MFB: *ACTA2, MYL9, TAGLN, EDNRA*) (**Figures 1D**, **S1** and **Table S2**). Among *PTPRC*/CD45^+^ cells we identified various myeloid cells including dendritic cells (DC-1: *CD1C, ITGAX*; DC-2: *LAMP3*), macrophages (MФ: *CD163*) and monocytes (non-classical monocytes MC-3 expressing *CD68, FCGR3A*; classical monocytes MC-1 and MC-2 expressing *CD14*). DC-1 also contained a minor population of *CD207^+^ CD1A^+^* Langerhans cells that did not cluster separately (**Figure 2D**). We also found a small cluster of B cells (BC: *MS4A1/*CD20) and NK/NKT-cells (*KLRC1*/NKG2, *KLRB1*/CD161, *NKG7*). One cluster (“other”) contained cells positive for *MALAT1*, suggesting damaged or dead cells (**Table S2**) (21). Among *CD3D^+^* T-cells, we found a large, distinct population of *CD4^+^* helper T-cells (TC-1, and adjacent TC-3 and TC-4) as well as two smaller clusters of *CD4^+^* helper and *CD8A^+^* cytotoxic T-cells (TC-2 and TC-5, respectively), with TC-2 also containing some *FOXP3^+^* regulatory T-cells, and TC-5 consisting of proliferating (*MKI67^+^*) cells (**Figures 2B, D**).

### Two T-Cell Clones Are Consistently Expanded in Skin, Blood and Lymph Node, Likely Derived From One Single Tumor Cell

**Figure 3:**
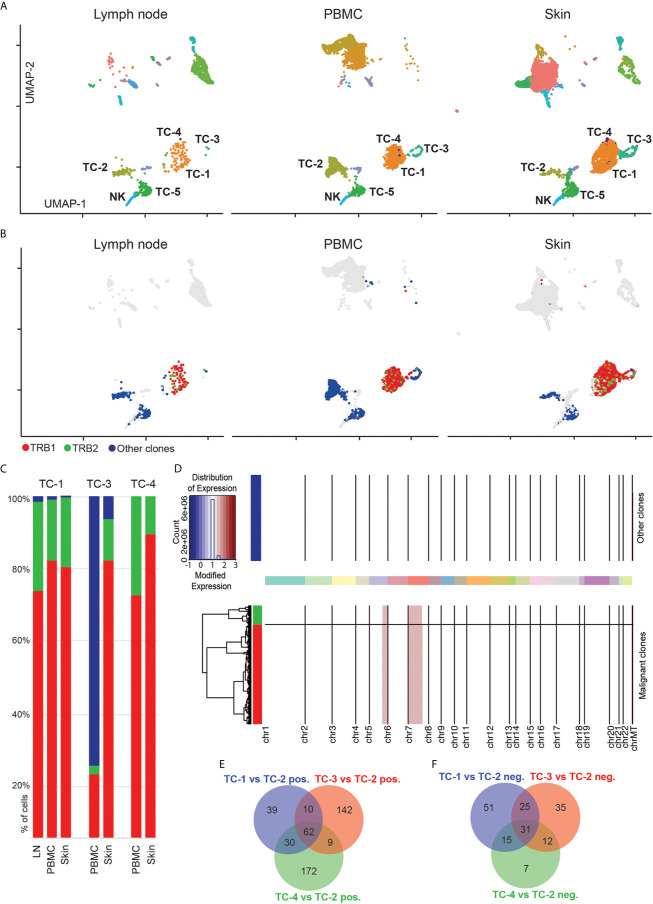
Expansion of two similar T-cell clones in blood, lymph node and skin. **(A)** Separate UMAP plots of lymph node, blood and skin samples; numbers refer to respective cell clusters. **(B)** Cells with detectable T-cell receptor are colored; cells without detectable T-cell receptor are displayed in grey. TCR+ polyclonal cells are labelled in blue, while the two top expanded clones are labelled in red and green, respectively. Data overlaid onto UMAP plots separately for each tissue. **(C)** Frequencies of most common clonotypes (red, green) and the polyclonal T-cell infiltrate (blue) in T-cell clusters TC-1, TC-3 and TC-4. **(D)** Copy number variation (CNV) analysis: Chromosomes are given on x-axis, and y-axis is proportional to cell numbers. Red indicates chromosomal gains. Dendrogram legend red is clonotype TRB1 and green TRB2. **(E, F)** Venn diagram of significantly up- and downregulated genes comparing tumor clusters TC-1, TC-3 and TC-4 with benign CD4+ T-helper cells from cluster TC-2 (adjusted p value<0.05, logFC>|0.5|). UMAP: Uniform Manifold Approximation and Projection; PBMC: Peripheral blood mononuclear cells.

When displaying cells separately for lymph node, blood and skin, we found most T-cell clusters to be present in all of these three body compartments (**Figure 3A**). By using 5’ scRNA-seq that allows simultaneous V-D-J sequencing of the T-cell receptor (TCR) alpha- and beta-chains (*TRA* and *TRB*), we overlaid *TCR*-positive cells onto UMAP plots (colored cells in **Figure 3B**). Generally, *TRA* and/or *TRB* sequences were detected in 2,952 cells which showed a polyclonal pattern in 45% of *TCR*-bearing cells (**Figure 3B**, labelled in blue, and **Table S3**). Similar to protein expression measured by immunohistochemistry (**Figures 1A, D**), the putative malignant cells expressed *CD3D* and *CD4* but lacked *CD5* and *CD7* (**Figures S2A, B**). Further analysis of the *TCR* sequences revealed two clones to be strongly expanded at 46% and 9% of all *TCR^+^* cells, which were confined within the *CD4^+^* T-cell clusters TC-1, TC-3 and TC-4 (top expanded clones marked in red and green, **Figure 3B**). The two TRB chains of these two clones differed by only one amino acid, namely *CASSQDRALENTIYF (“TRB1”)* and *CASSQDRTLENTIYF (“TRB2”)*, due to a single nucleotide change (guanine to adenine). These *TRB* chains were mainly paired with the *TRA* chain *CAVDHARLMF*, and in <5% of clonal cells also with *CALSKKPGRKAYLRT* (**Table S3**). TC-1 in skin contained 79% *TRB1^+^* and 20% *TRB2^+^* cells, with comparable distributions in blood (*TRB1*: 82%, *TRB2*: 17%) and lymph node (*TRB1*: 73%, *TRB2*: 26% of all cells) within this cluster (**Figure 3C**). The much smaller cluster TC-4 was sufficiently present only in skin and blood, with roughly the same distribution of *TRB1^+^* and *TRB2^+^* cells. In cluster TC-3, only skin showed largely clonal *TRB1* and *TRB2* percentages, while blood cells were mostly polyclonal (**Figures 3B, C**).

The expression of two distinct *TRB* within the clonally expanded T-cell population either represents spontaneous mutation from one single clone, or the presence of two independent clones (22). When comparing gene expression of cells harboring *TRB1* versus *TRB2*, we did not detect any significant differences (at an adjusted p<0.05, data not shown). Furthermore, we also used inferCNV to identify copy number variations between these two populations (23). Also on this level we did not find relevant differences between *TRB1* and *TRB2* clonotypes, but identified similar gains on chromosomes 5 and 7 in both clones (**Figure 3D** and **Table S4**), the latter being consistent with observations in previous CTCL whole exome sequencing studies (24). Taken together, these findings suggest that both expanded clones have a common T-cell ancestor. Based on these observations we conclude that clusters TC-1, TC-4 and in part TC-3 represent the malignant CTCL clone, while clusters TC-2 and TC-5 harbor benign helper and cytotoxic T-cells, respectively. To further define characteristics of these cells, we calculated differentially expressed genes between polyclonal, presumably benign *CD4^+^* helper T-cells (TC-2) and clusters TC-1, TC-3 and TC-4 (**Table S5**). We observed substantial transcriptomic differences between these three clusters (**Figures 3E, F**, and **Table S6**). Cluster TC-3 contained mostly proliferating cells, as evidenced by the upregulation of *MKI67* (**Figure 2D** and **Table S5**). Despite consistent *CD3D* expression in cluster TC-4, most cells co-expressed markers typically found in myeloid cells such as *CD14, CD68*, and *CD1C* (**Table S5**), and overall RNA content was approximately doubled in TC-4 when compared to other, non-proliferating cells (**Figures S2C, D**), suggesting either the presence of technical doublets, or the engulfment of T-cells by professional phagocytes (25), a question that cannot be definitively answered with the current dataset.

### Malignant Cells Display Markers of Dermal Tissue Resident Memory T-Cells, and Simultaneously Express Th2, Th17 and Th22-Associated Cytokines in a Tumor-Permissive Microenvironment

**Figure 4:**
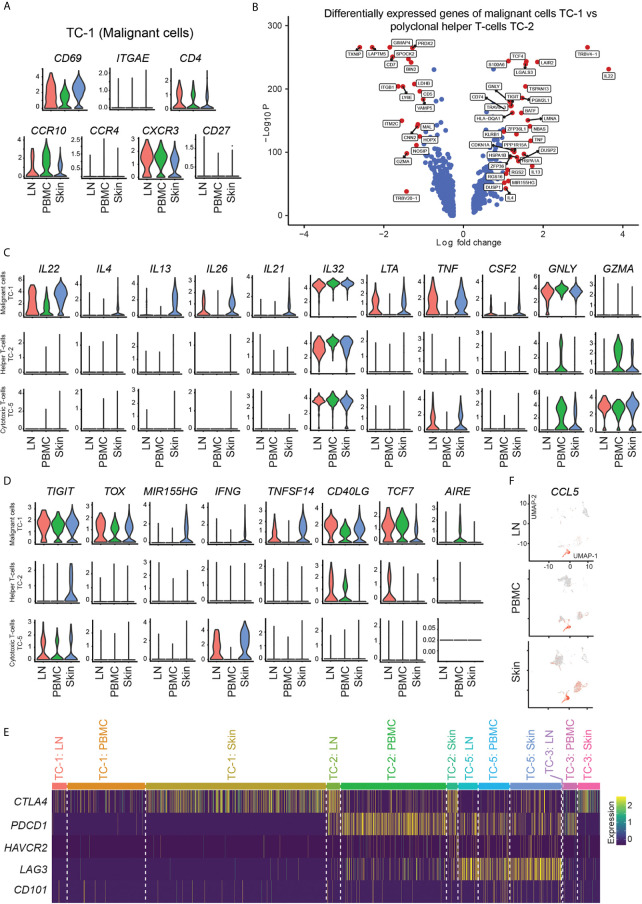
Marker expression of malignant and benign T-cells. **(A)** Violin plots of T-cells in cluster TC-1 showing distribution of normalized gene expression levels of the respective genes in lymph node (red), blood (green) and skin samples (blue). **(B)** Volcano plot of differentially express genes between T-cells in malignant cluster TC-1 and benign T-helper cell cluster TC-2 combining all samples as calculated by Wilcoxon Rank Sum Test and Bonferroni correction. Genes with log fold change >|1.0| are labeled in red. **(C, D)** Violin plots of TC-1 (clonally expanded tumor cells), TC-2 (benign helper T-cells) and TC-5 (benign CD8+ cytotoxic T-cells) showing the distribution of normalized gene expression levels of the respective genes in lymph node (red), blood (green) and skin samples (blue). **(E)** Expression heat map of selected checkpoint molecules. Upregulation is indicated in yellow, downregulation in purple. **(F)** Feature plots of CCL5 (RANTES). Normalized expression levels for each cell are color-coded (red) and overlaid onto separate UMAP plots for lymph node (LN), blood (PBMC) and skin. Intensity red color reflects respective level of expression.

Malignant cells in MF are thought to be closely related to tissue-resident memory T-cells (T_RM_), given many phenotypic similarities (26). In our patient, clonally expanded cells in cluster TC-1 were *CD4^+^ CD69^+^ ITGAE/*CD103^-^ (**Figure 4A**) suggesting their close relation to dermal, but not epidermal T_RM_ (27). In addition, these cells expressed the skin homing molecule *CCR10*, but only weakly *CCR4* (**Figure 4A**). Another chemokine receptor, *CXCR3*, was specifically expressed in cells of cluster TC-1 (**Figure 4A**), but was hardly present in benign helper T-cells of cluster TC-2 (data not shown). In cells from TC-1, representing the largest cluster of malignant cells, we found increased expression of various cytokines in comparison to benign helper T-cells from cluster TC-2, including type 22 (*IL22*), type 2 (*IL4, IL13*), and type 17 (*IL26*) cytokines, as well as *IL21* and *IL32* (**Figures 4B, C**, **Table S5**). In addition, they were rich in *LTA, TNF, CSF2*, and *GNLY* (**Figure 4C** and **Table S5**), creating a highly inflammatory environment that could not be attributed to a single classic T helper cell subset (28). We also found increases in the T-cell exhaustion-associated markers *TIGIT* and the CTCL-associated markers *TOX* (29) and *MIR155* (30) (**Figure 4D**). *IL22, IL32* and *GNLY*, as well as *TOX* and *TIGIT* were generally increased in skin, blood and lymph nodes, when compared to benign helper T-cells or cytotoxic T-cells (**Figures 4C, D**), consistent with a pro-inflammatory malignant phenotype spanning several body compartments (**Table S7**). By contrast, Th2 cytokines (*IL4* and *IL13*) and associated mediators (*IL21*, *TNFSF14*/LIGHT) (31, 32), but also *IFNG* and *MIR155* were mostly upregulated in malignant cell from skin (**Figures 4C, D**, **Figures S3A, B** and **Table S8**). The Th17-associated cytokine *IL26*, colony-stimulating factor-2 (*CSF2*), the co-stimulatory molecule *CD40LG* and tumor necrosis factor-alpha (*TNF*) were upregulated both in lymph nodes and skin, but less so in blood (**Figures 4C, D**). Expression of the transcription factors *AIRE* (previously associated with negative selection of self-recognizing T-cells) (33) and *TCF7* (associated with central memory T-cells) (34) discriminated the malignant cells in peripheral blood from those in skin (**Figure 4D**). *CD27*, previously described to be absent from terminally differentiated memory T-cells (35), was universally downregulated in lymphoma cells (**Figure 4A**). Taken these data together, we found that malignant clones harbored a multitude of pro-inflammatory mediators, in stark contrast to benign T-cells.

Next, we assessed factors involved in the control of cell activation, namely inhibitory receptors such as *CTLA4, PDCD1* (PD-1), *HAVCR2* (TIM-3), *LAG3*, and *CD101* (IGSF2) (36). In line with their strong pro-inflammatory phenotype, clonally expanded cells showed absence of these markers, with only a partial expression of *CTLA4* (**Figure 4E**). By contrast, benign helper T-cells (TC-2) expressed *PDCD1* and partly *LAG3* and *CTLA4*, while cytotoxic T-cells (TC-5) highly expressed *LAG3* (**Figure 4E**), consistent with their less inflammatory phenotype. These data indicate that the lack of inhibitory molecules might be involved in the aberrant inflammatory cytokine pattern of clonally expanded cells, suggesting a cell-intrinsic contribution of hyperactivation in these cells.

Besides the malignant clone, benign inflammatory cells surrounding CTCL cells have also been assumed to play a role in control versus progression of the disease (6). Activated CD8^+^ cytotoxic T-cells have been postulated as major anti-CTCL cells in early lesions when the disease is still confined to the skin, but lose this capacity when the disease progresses (6). *CD8A^+^* cells in cluster TC-5 were rich in the killer molecules *GZMA* (**Figure 4C**), *GZMK, GZMH*, and *GZMB* (**Figure S1**), and were main producers of *IFNG* in skin and lymph nodes (**Figure 4D** and **Table S2**), the type-1 lead cytokine associated with anti-CTCL properties (37). However, *CD8A^+^* cells also ubiquitously showed highest levels of *CCL5* (RANTES) (**Figure 4F** and **Table S2**), a chemokine previously shown to maintain CD4^+^ T_RM_ cells after infection or sensitization (38), but that also attracts monocytes which were shown to promote the survival of MF cells in a mouse model (39). NK/NKT-cells were also rich in *GZMA, GZMB, CCL5* and *IFNG* (**Figure S1** and **Table S2**), but did not show significant differences between skin and lymph nodes (**Table S9**). Benign helper T-cells (TC-2) displayed significantly increased levels of *IL2RA* and *TNFRSF18* (GITR) in skin compared to lymph node tissue (**Figure S3C** and **Table S10**), consistent with a more regulatory phenotype. Compared to blood, they showed increases in the checkpoint molecule *CTLA4*, most likely due to increased levels of *FOXP3^+^* regulatory T-cells within this cluster (**Figure S3D** and **Table S10**). Conversely, levels of the cytotoxic molecule *TNFSF10* (TNF-related apoptosis-inducing ligand TRAIL) were decreased compared to blood cells (**Figure S3D** and **Table S10**), potentially marking decreased anti-tumor activity (40). Benign skin versus blood helper T-cells also showed increases in *ICOS* (CD278) (**Figure S3D** and **Table S10**), a costimulatory molecule present in activated T-cells especially associated with type 2 inflammation (41), corroborating a more Th2 versus Th1-skewed skin phenotype, consistent with CTCL progression (6, 42). These data suggest that both, helper and cytotoxic T-cells surrounding CTCL cells in skin, have several regulatory features and may thus help in sustaining a pro-tumorigenic environment.

### Myeloid Cells Exhibit Markers Consistent With a Type 2 and Regulatory Immune Phenotype

**Figure 5:**
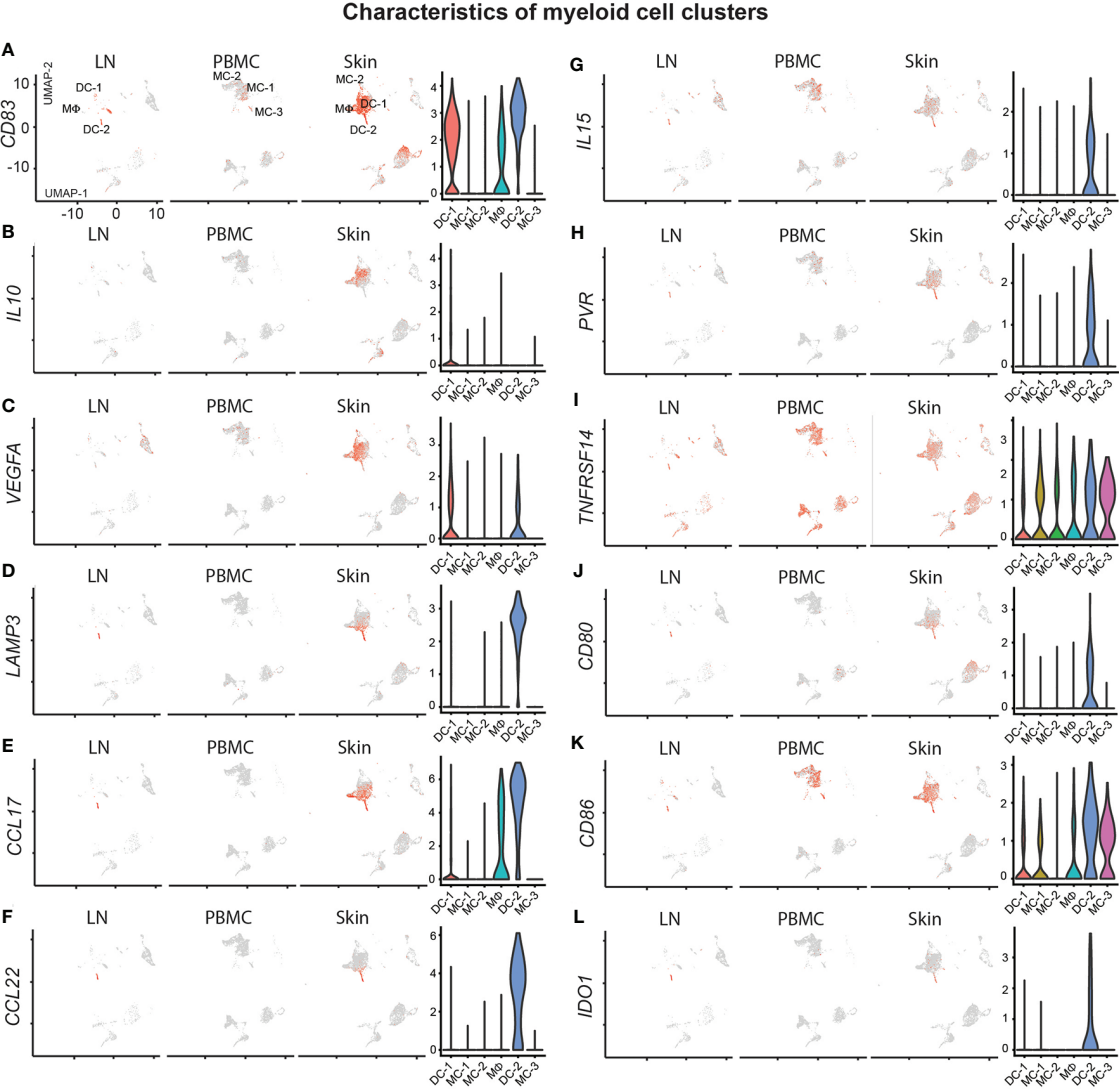
Characterization of myeloid cell clusters. **(A–L)** For feature plots, normalized expression levels for each cell are color-coded (red) and overlaid onto separate UMAP plots for lymph node (LN), blood (PBMC) and skin. Intensity of color reflects respective level of expression. Violin plots of the combined samples show the distribution of normalized gene expression levels of the respective genes in color-coded DC-1, MC-1, MC-2, MΦ, DC-2 and MC-3 clusters.

Myeloid cells, including dendritic cells, play a central role in T-cell biology, including *in vitro* survival of malignant T-cells (39). We found a large population of *ITGAX*/CD11c dendritic cells (DC-1) in skin, strongly positive for the Th2-associated markers amphiregulin (AREG), and the chemokines *CXCL2, CXCL3* and *CXCL8* (**Figure S1** and **Table S2**). While previous reports suggest an abundance of immature dendritic cells in advanced CTCL lesions implicated in tumor progression (39), DC-1 highly expressed the maturation marker *CD83* (**Figure 5A**). Nevertheless, they were the main producers of the regulatory cytokine *IL10* (**Figure 5B**), and positive for *VEGFA* (**Figure 5C**), a major angiogenetic growth factor associated with advanced CTCL (43).

Dendritic cells from cluster DC-2 harbored the maturation marker *LAMP3* (**Figure 5D**) and were present only at relatively small frequencies in comparison to all other myeloid cells (**Figure 2B**), but were also found in lymph node tissue, consistent with previous reports (44). They were characterized by peak expression of Th2-associated chemokines (45) *CCL17* and *CCL22* (**Figures 5E, F**). DC-2 also expressed *IL15* both in skin and lymph node tissue (**Figure 5G**), a cytokine previously shown to promote CTCL (46) and implicated in the maintenance of T_RM_ (47), as well as the receptors for tumor cell derived *TIGIT, TNFSF14* (LIGHT) and *CTLA4*, namely *PVR, TNFRSF14* and *CD80/CD86*, respectively (**Figures 5H–K**). They also expressed *IDO1* (**Figure 5L**), coding for the enzyme indoleamine-pyrrole 2,3-dioxygenase that has immunosuppressive and T-cell modulatory functions, potentially involved in CTCL pathogenesis (48).

Skin macrophages (MФ) were also positive for Th2-promoting chemokines such as *CCL18, CCL13* and *CCL17* (**Figure S1**, **Figure 5E** and **Table S2**). Blood monocytes in clusters MC-1, MC-2 and MC-3 were found only at traces in lymph node and skin tissues, and generally lacked chemokine expression or activation markers such as CD83 (**Figure 5A**). Taken together, myeloid cells were largely skewed towards more type 2 inflammation and regulatory mediators, consistent with a more pro-CTCL tumor microenvironment (6).

### Trajectory Inference Reveals Transcriptomic Heterogeneity Within the Malignant Clone, Reflecting Differences in Inflammatory and Migratory Properties

**Figure 6:**
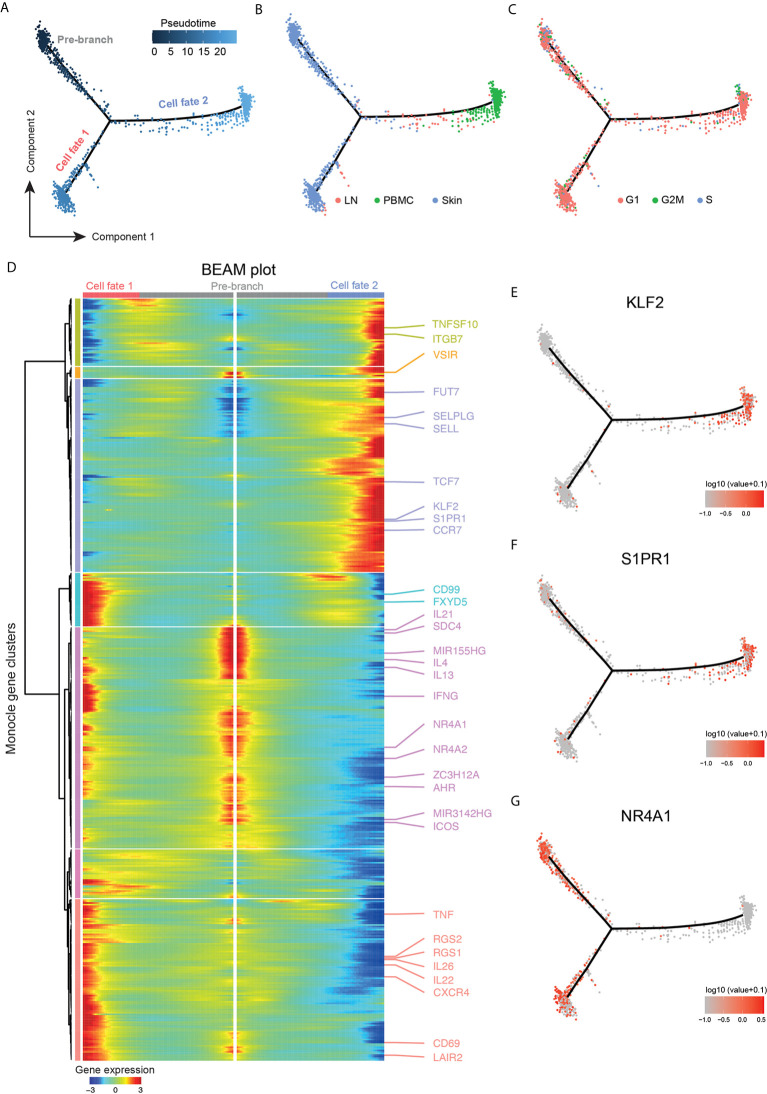
Trajectory analysis highlighting tumor clone plasticity throughout body compartments. **(A)** Pseudotime ordering of malignant cells from clusters TC-1 and TC-3 along a bifurcated cell trajectory. Plot colored according to pseudotime. **(B)** Trajectory plot colored according to tissue of origin in red (LN: lymph node), green (PBMC, peripheral blood mononuclear cells), and blue (skin). **(C)** Trajectory plot colored according cell cycle phase in red (G1), green (G2/M) and blue (S). **(D)** Bifurcated expression heat map of genes for bifurcation between cell fates 1 and 2. Middle of heat map shows pre-branch cells, left side the expression of cells from “cell fate 1”, and right side the expression blood-oriented cells (“cell fate 2”). **(E–G)** Trajectory plots with overlaid expression of respective genes, highest expression in red, lowest expression in grey.

To gain further insight into the relationship of cellular fates in skin, blood and lymph node (19), we applied trajectory inference analysis using the Monocle 2 algorithm on malignant T-cells (**Figure 6A**). In the resulting trajectory, cells from peripheral blood and skin were found at opposing ends, while lymph node cells were mostly scattered along the manifold (**Figure 6B**), suggesting transcriptomic properties related both to skin and blood cells.

The influence of cell cycle genes in proliferating cells was not the major determinant for pseudotime alignment since cycling cells were found on all branch ends, and were spread throughout the trajectory (**Figure 6C**). To discern genes responsible for the construction of the manifold, branched expression analysis modeling (BEAM) was performed (**Figure 6D**). Genes with a *q*-value<10^-5^ were considered for further analyses. For the main branching (“cell fate 1” versus “cell fate 2”), 500 genes fulfilled this criterion (**Table S11**). There was also a small side branch within “cell fate 1” containing a few lymph node cells (**Figure 6B**), but it did not elicit significantly regulated genes (all *q*-value>0.1) and was therefore not further analyzed (data not shown). In blood, the transcription factor *KLF2* as well as the target downstream gene *S1PR1* were upregulated (**Figures 6D–F**), consistent with the loss of tissue retention (49). Concordantly, the transcription factor *TCF7*, which is usually downregulated on T_RM_ and is important for T_CM_ differentiation (49), was expressed on malignant cells in the blood, together with the T_CM_ surface markers and lymph node homing receptors *SELL* and *CCR7* (**Figure 6D**).

Importantly, malignant cells of the peripheral blood expressed the skin-homing receptor CLA as shown by flow cytometry (**Figure S4**), consistent with corresponding gene upregulation of both *SELPLG* and the fucosyltransferase *FUT7* (50) (**Figure 6D**), indicating their retained capability to home to the skin (51, 52).

*NR4A1* (Nur77), *NR4A2*, and *AHR*, markers associated with tissue retention of T_RM_ cells, as well as the CTCL-typical micro-RNAs *MIR155* and *MIR3142* were all associated with the skin phenotype (**Figures 6D, G**). “Pre-branch” cells expressed Th2-associated mediators (*IL4, IL13, IL21, ICOS*). Cells in the skin after the bifurcation (“cell fate 1”) further upregulated transcripts characteristic of T_RM_ including *CD69, CXCR4* (53), and regulator of G protein signaling 1 and 2 *(RGS1, RGS2)*, and these cells showed additional upregulation of Th22, TH17 and Th1-associated cytokines (*IL22, IL26, TNF, IFNG*), while “pre-branch” cells expressed several immunoregulatory markers such as *VSIR, SDC4*, and *ZC3H12A.* In addition, “cell fate 1” cells showed upregulation of transcripts involved in cell motility including *LAIR2* (54)*, CD99* (55) and *FXYD5* (56), discriminating them from “pre-branch” cells. These data support intra-tumor heterogeneity not only between tissues, but also among malignant cells of the skin, reflected by distinct profiles of regulatory, inflammatory and potentially migratory properties.

## Discussion

Reliable tracing of individual T-cell populations in humans can be a major challenge. scRNA-seq with simultaneous V-D-J sequencing of the TCR now allows for the investigation of specific T-cell clones and their transcriptomic behavior throughout various body compartments. In our patient, malignant cells of the skin showed all the characteristics of benign T_RM_ cells as described before, with expression of the skin homing molecule *CCR10* as well as *CD69* accompanied by downregulation of the transcription factor *KLF2* (57), and ample production of cytokines (58). In blood, by contrast, these MF cells showed a loss of the tissue retention signature as evidenced by the upregulation of *KLF2* and, consequently, upregulation of *S1PR1* and downregulation of *CD69*, indicative of a shift towards a more T_CM_-like phenotype, consistent with increased *TCF7* expression (57). In accordance, lymph node homing receptors (*SELL* and *CCR7*) were upregulated. Until recently, T_RM_ were believed to be a sessile, non-recirculating, terminally differentiated population restricted to their non-lymphoid tissue of residence, such as the skin (59, 60). This dogma has recently been challenged by observations in mice showing equilibration of skin resident memory T-cells upon parabiosis over 12-16 weeks (38). Importantly, the malignant clone in our patient displayed exactly the same shift in phenotype as observed in T_RM_ in a murine model, demonstrating that CD8^+^ T_RM_ from skin can rejoin the circulation after antigenic stimulation or activation (61). They could then either maintain their phenotype and home back to their tissue of origin, or even differentiate into T_EM_ and T_CM_ cells (61). In a xenograft mouse model, T_RM_ cells had the capability to migrate out of the skin, but showed preferential homing back to a human skin equivalent (62). In line, blood CD4^+^CD69^-^CD103^+^ cells were transcriptionally and clonally related to skin CD4^+^CD69^+^CD103^+^ T_RM_ in this model system (62). In our MF patient, malignant cells in blood seemed to also retain their skin-homing capabilities, indicated by maintained CLA and *CCR10* expression. The systemic dissemination of tumor cells in MF could therefore reflect the physiological migratory behavior of tissue resident memory T-cells, and might help to explain the clinical observation that MF lesions can spread to virtually any skin site, which, intuitively, needs to happen via the bloodstream. Importantly, it is not yet understood which factors regulate the egress or the reentry of T_RM_ from non-lymphoid tissues. Upregulation of certain pro-migratory mediators (*LAIR2, CD99, FXYD5*) by a subset of skin lymphoma cells might contribute to the altered migratory behavior of these cells, but this hypothesis needs corroboration in functional studies.

T_RM_ cells are poised to rapidly react after antigenic re-challenge, a process that is assumed to be under stringent control by inhibitory receptors (36), that can also be present on CTCL cells (5). In our patient, these receptors were largely absent, except for *CTLA4*. Secreted CTLA4 can act on dendritic cells inducing the expression of IDO and rendering them tolerant (63). Additionally, the resulting tryptophan deprivation and generation of toxic metabolites have been shown to preferentially induce apoptosis in Th1 cells and less so in Th2 cells, which would favor a pro-tumorigenic environment (6). In our patient, a distinct population of LAMP3^+^ dendritic cells expressed the CTLA4 receptors *CD80* and *CD86*, as well as *IDO1*. They also expressed *TNFRSF14* and *PVR*, the receptors for *TNFSF14* (LIGHT) and *TIGIT*, respectively, which are both synthesized by the tumor cells, and might additionally enhance local Th2 immune skewing (31, 64). The main population of skin myeloid cells (DC-1) were *CD83^+^* co- expressing *VEGFA* and small amounts of *IL10*, features that are implicated in the sustainment of a pro-tumorigenic milieu (65, 66).

This study is limited by data derived from only one patient, at one single (advanced) stage of disease. Also, due to scarcity of sample, we only had few lymph node cells available. Given the substantial heterogeneity not only between CTCL patients, but also within the malignant clone (11 *,* 67 *,* 68), our findings need corroboration in additional patients and subtypes of CTCL. Nevertheless, the general possibility to trace a defined clone throughout three human body compartments sheds new light on the transcriptomic plasticity within these cells, which might be directly linked to their migratory behavior. Future studies will need to clarify the role of cell intrinsic mechanisms versus the impact of the tissue microenvironment in this regard.

## Data Availability Statement

The datasets presented in this study can be found in online repositories. The names of the repository/repositories and accession number(s) can be found below: https://www.ncbi.nlm.nih.gov/geo/, GSE165623.

## Ethics Statement

The studies involving human participants were reviewed and approved by Ethics Committee of the Medical University of Vienna, Austria. The patients/participants provided their written informed consent to participate in this study.

## Author Contributions

Conceptualization, PB, MF, WB, CJ, KR. Software and Formal Analysis, KR, MW. Investigation, MF, WB, LS, CJ, ISK, MM, FT, SP. Writing – Original Draft, WB, CJ, KR, WW. Writing – Review and Editing, PB, MF, KR. Visualization, TR. Funding Acquisition, PB. All authors contributed to the article and approved the submitted version.

## Funding

This work was funded by a research grant to PMB from the Austrian Science Fund (grant number KLI 849-B). MF was supported by a research grant from the Austrian Science Fund (grant number SFB-F61.03).

## Conflict of Interest

CJ is an employee of the Medical University of Vienna, and has received personal fees from AbbVie, Almirall, Amgen, Eli Lilly, Janssen, LEO Pharma, Mallinckrodt/Therakos, Pfizer, Novartis, Sandoz, Takeda, and UCB. CJ is an investigator for Eli Lilly and Company, Novartis and 4SC (grant paid to her institution). PB is an employee of the Medical University of Vienna, and has received personal fees from LEO Pharma, Pfizer, Sanofi Genzyme, Eli Lilly, Novartis, Celgene, UCB Pharma, Biotest, Boehringer Ingelheim, AbbVie, Amgen and Arena Pharmaceuticals. PB is an investigator for Novartis (grant paid to his institution). WB is an employee of the Medical University of Vienna and has received personal fees from Takeda, Abbvie, GSK/ViiV and Gilead. SP has received personal fees from Takeda. MM has received personal fees (speaker honoraria) from Siemens and Bristol Myers Squibb. WW is an employee of the Medical University of Vienna and has received personal fees from LEO Pharma, Pfizer, Sanofi Genzyme, Eli Lilly, Novartis, Boehringer Ingelheim, AbbVie, and Janssen.

The remaining authors declare that the research was conducted in the absence of any commercial or financial relationships that could be construed as a potential conflict of interest.

## Supplementary Material

Supplementary Figure 1 Cluster marker heat map. Heat map displaying the top 10 differentially expressed genes (according to highest log-fold change ordered by smallest adjusted p value using Wilcoxon Rank Sum Test with Bonferroni correction for each cluster compared with the rest of the dataset); upregulation is indicated in yellow, and downregulation in purple; numbers on top refer to the respective cell clusters displayed in **Figure 2B**.

Supplementary Figure 2
**(A, B)** Feature plots of CD5 and CD7, respectively. Normalized expression levels for each cell are color-coded (red) and overlaid onto separate UMAP plots for lymph node (LN), blood (PBMC) and skin. Intensity of red color reflects respective level of expression. **(C)** Violin plots showing the number of genes per cell, the percentage of mitochondrial gene content and counts per cell in all clusters. **(D)** Violin plots showing the computed doublet score for each cluster combined for all samples.

Supplementary Figure 3 Volcano plots of differentially expressed genes comparing skin and lymph node or skin and PBMCs in **(A, B)** TC-1 and in **(C, D)** TC-2, as calculated by Wilcoxon Rank Sum Test and Bonferroni correction. PBMC: Peripheral blood mononuclear cells.

Supplementary Figure 4 Flow cytometric analysis of PBMCs confirming CLA expression of circulating MF tumor cells (CD3+CD4+CD7-). Blue histogram denotes isotype control.

Supplementary Table 1 Cell counts per cluster and sample derived from our scRNA-seq dataset.

Supplementary Table 2 Top 10 differentially expressed genes (scRNA-seq clustermarker) according to highest log fold change ordered by smallest adjusted p-value using Wilcoxon Rank Sum Test with Bonferroni correction for each cluster, as compared to the rest of the dataset in all sequenced skin cells.

Supplementary Table 3 Numbers and frequencies of top 20 TCR clones as detected by TRA and/or TRB expression.

Supplementary Table 4 Regions with copy number variations in malignant cells with clonotype TRB1 and TRB2. State 4= addition of one copy.

Supplementary Table 5 Differential gene expression analysis within cell types of scRNA-seq analyses as defined by logFC>|0.25| and adjusted p-value<0.05, using Wilcoxon Rank Sum Test and Bonferroni correction. Comparisons of cluster TC-2 vs cluster TC-1, cluster TC-2 vs cluster TC-3, and cluster TC-2 vs cluster TC-4 are shown.

Supplementary Table 6 Regulated genes (differentially expressed genes) corresponding to the Venn diagram in **Figures 3E, F**.

Supplementary Table 7 Top 100 differentially expressed genes (scRNA-seq clustermarker) for T-cell clusters, according to highest log fold change ordered by smallest adjusted p-value using Wilcoxon Rank Sum Test with Bonferroni correction, as compared to the rest of the dataset, calculated separately for skin, blood and lymph node (LN) samples.

Supplementary Table 8 Differential gene expression analysis within cell types of scRNA-seq analyses as defined by logFC>|0.25| and adjusted p-value<0.05, using Wilcoxon Rank Sum Test and Bonferroni correction. Comparison of lymph node (LN) vs skin, and skin vs blood (PBMC) samples within cluster TC-1.

Supplementary Table 9 Differential gene expression analysis within cell types of scRNA-seq analyses as defined by logFC>|0.25| and adjusted p-value<0.05, using Wilcoxon Rank Sum Test and Bonferroni correction. Comparison of lymph node (LN) and skin samples within the NK cell cluster.

Supplementary Table 10 Differential gene expression analysis within cell types of scRNA-seq analyses as defined by logFC>|0.25| and adjusted p-value<0.05, using Wilcoxon Rank Sum Test and Bonferroni correction. Comparison of lymph node (LN) vs skin, and skin vs blood (PBMC) samples within cluster TC-2.

Supplementary Table 11 Genes with branch dependent changes in gene expression according to branched expression analysis modeling (BEAM) from the R package Monocle. Only genes with a *q*-value <10^-6^ and expressed in more than 10 cells are shown in the table and in **Figure 6D** (500 genes).

## Abbreviations

CTCL, Cutaneous T-cell lymphoma; MF, Mycosis fungoides; mSWAT, Modified Severity Weighted Assessment Tool; TCR, T-cell receptor; T_CM_, Central memory T-cell; T_EM_, Effector memory T-cell; T_RM_, Tissue resident memory T-cell; scRNA-seq, Single-cell RNA sequencing; TRA, T-cell receptor alpha locus; TRB, T-cell receptor beta locus; CDR3, Complementarity-determining region 3.
