# Supplementary figures and images for: Single-Cell RNA Sequencing Reveals Tissue Compartment-Specific Plasticity of Mycosis Fungoides Tumor Cells
Source: Front Immunol. 2021 Apr 21;12:666935. doi: 10.3389/fimmu.2021.666935 (PMC8097053; doi:10.3389/fimmu.2021.666935)

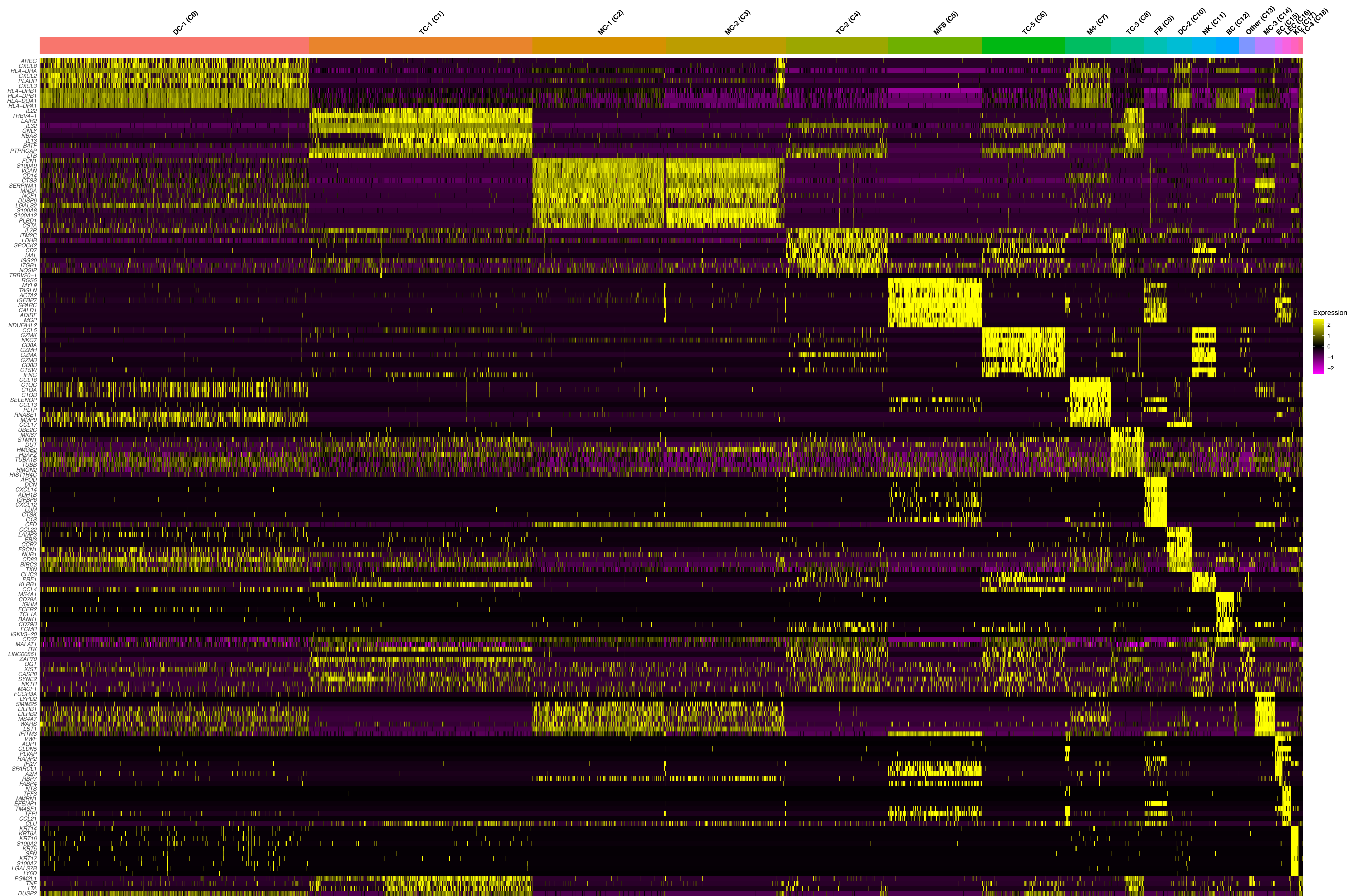

Supplement: Supplementary Figure 1 — Cluster marker heat map. Heat map displaying the top 10 differentially expressed genes (according to highest log-fold change ordered by smallest adjusted p value using Wilcoxon Rank Sum Test with Bonferroni correction for each cluster compared with the rest of the dataset); upregulation is indicated in yellow, and downregulation in purple; numbers on top refer to the respective cell clusters displayed in Figure 2B . [file Image_1.pdf]

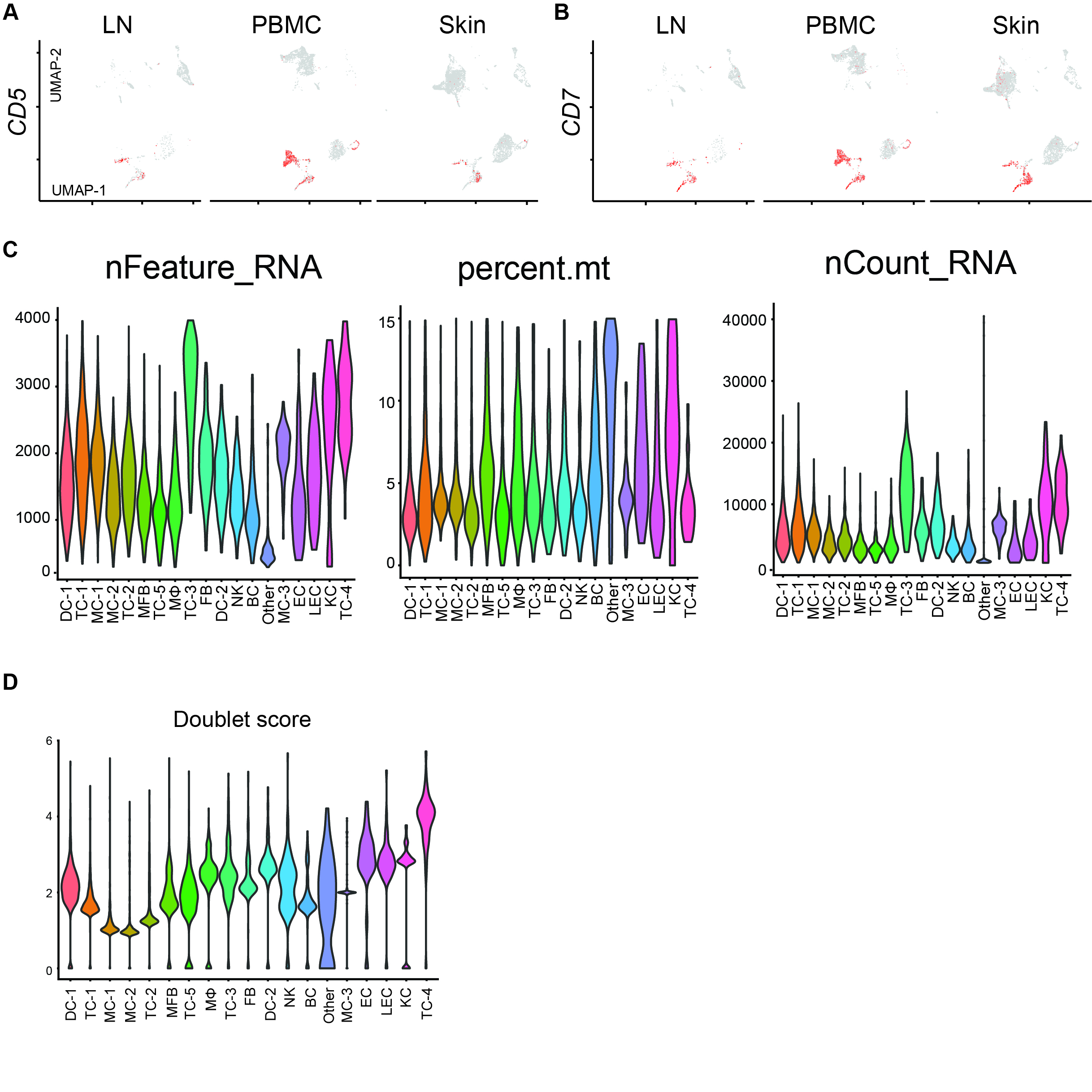

Supplement: Supplementary Figure 2 — (A, B) Feature plots of CD5 and CD7, respectively. Normalized expression levels for each cell are color-coded (red) and overlaid onto separate UMAP plots for lymph node (LN), blood (PBMC) and skin. Intensity of red color reflects respective level of expression. (C) Violin plots showing the number of genes per cell, the percentage of mitochondrial gene content and counts per cell in all clusters. (D) Violin plots showing the computed doublet score for each cluster combined for all samples. [file Image_2.jpeg]

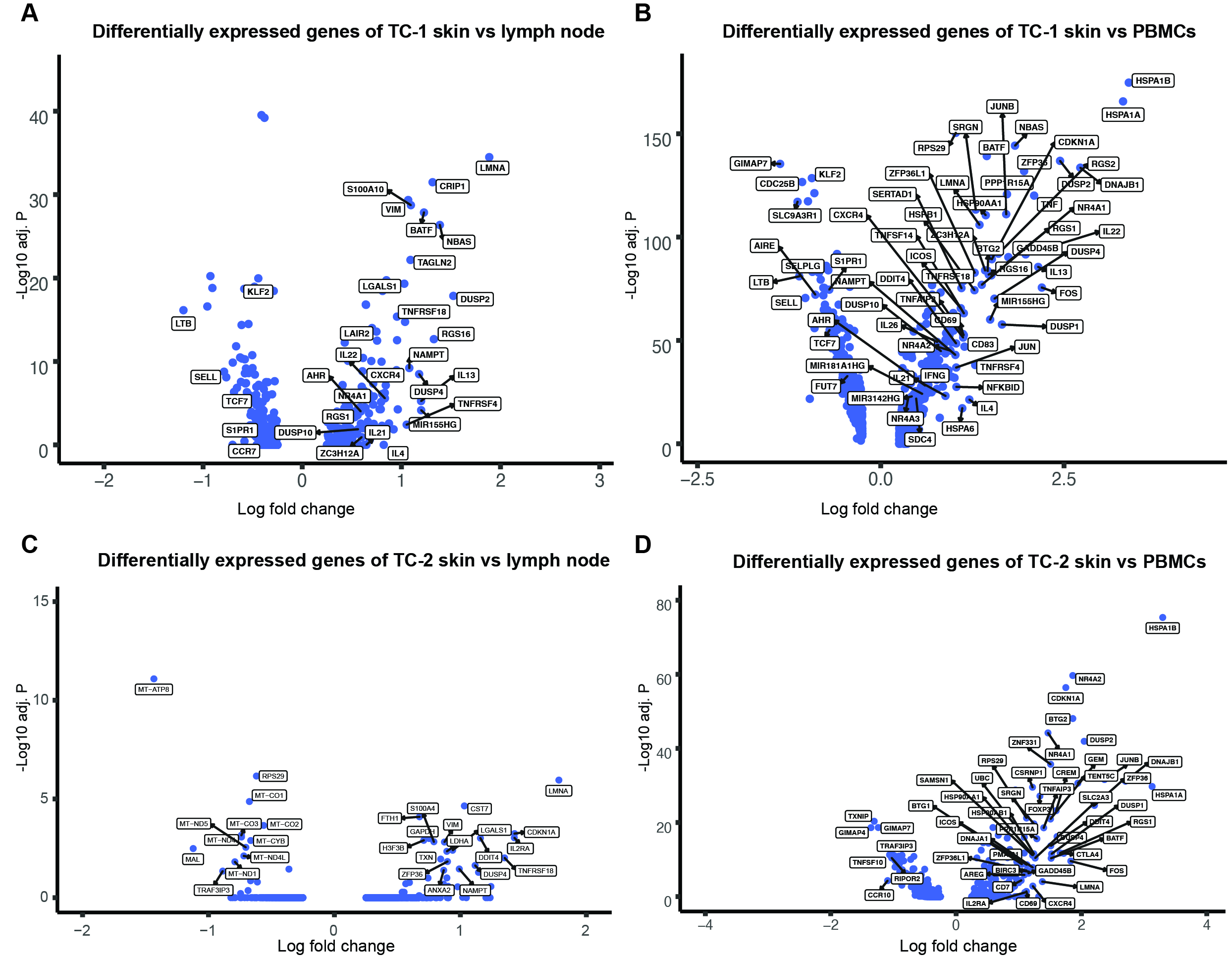

Supplement: Supplementary Figure 3 — Volcano plots of differentially expressed genes comparing skin and lymph node or skin and PBMCs in (A, B) TC-1 and in (C, D) TC-2, as calculated by Wilcoxon Rank Sum Test and Bonferroni correction. PBMC: Peripheral blood mononuclear cells. [file Image_3.jpeg]

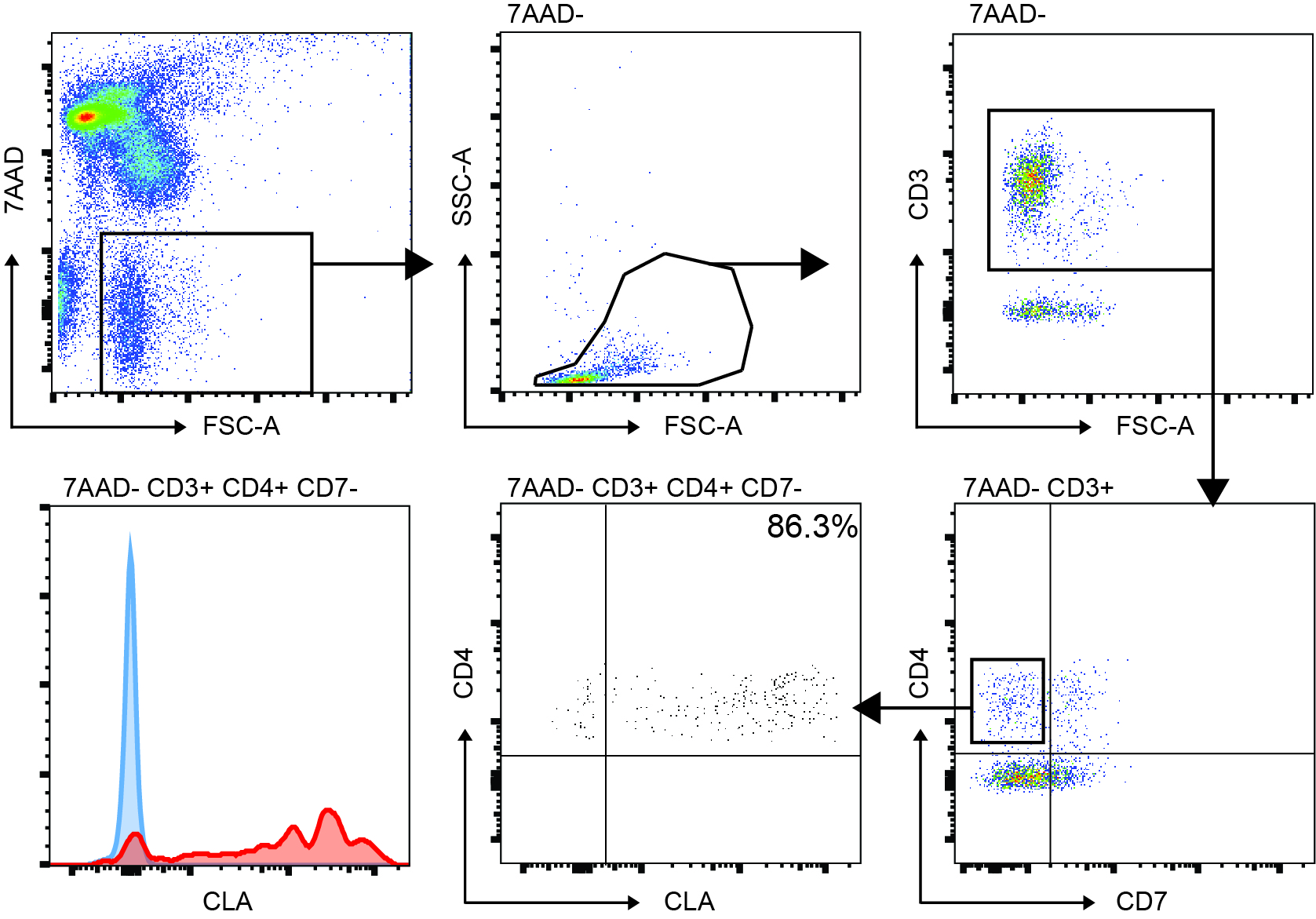

Supplement: Supplementary Figure 4 — Flow cytometric analysis of PBMCs confirming CLA expression of circulating MF tumor cells (CD3+CD4+CD7-). Blue histogram denotes isotype control. [file Image_4.jpeg]
